# Tailor-made biocatalysts: combining thermodynamics, organic synthesis, molecular biology, biochemistry and microbiology for the design of enzyme selections

**DOI:** 10.5936/csbj.201209013

**Published:** 2012-10-28

**Authors:** Jean-Luc Jestin

**Affiliations:** a Unité de Virologie Structurale, Département de Virologie, Institut Pasteur, 25 rue du Dr Roux, 75724 Paris Cedex 15, France

## Abstract

A general strategy for the isolation of catalysts for given chemical reactions was designed.

A first link between genes and their corresponding proteins was established by phage display: using Darwin's principles on evolution based on selection and amplification, rare protein molecules can then be selected for function from a large repertoire prior to their characterization by sequencing of their genes.

A second link was created between enzymes and their products. By making use of the chelate effect and of *Inovirus* particles as a chemical, affinity chromatography for the reaction product is then sufficient to isolate among 10^6^ to 10^11^ proteins and their genes, the rare ones coding for catalysts of interest. The strategy for the parallel processing of information on the catalytic activity of variants from a large protein repertoire is highlighted in this review.

## Introduction

The search of catalysts for chosen chemical reactions is an old challenge in chemistry. It may even be stated that the challenge is at least as old as chemistry: a major aim of alchemists was indeed the finding of a philosopher's stone so as to convert various metals into gold. The introduction of logic and rational thought in the history of knowledge in the 17th century allowed contemporary scientific approaches. The synthesis of urea by F. Woehler in 1828 is often regarded as the birth of organic chemistry. While organic chemistry may be viewed as a discipline which is more than 180 years old, it is only within the two last decades that experimental strategies are sufficiently general to address the problem of the identification of catalysts for given chemical reactions.

**Scheme 1:**
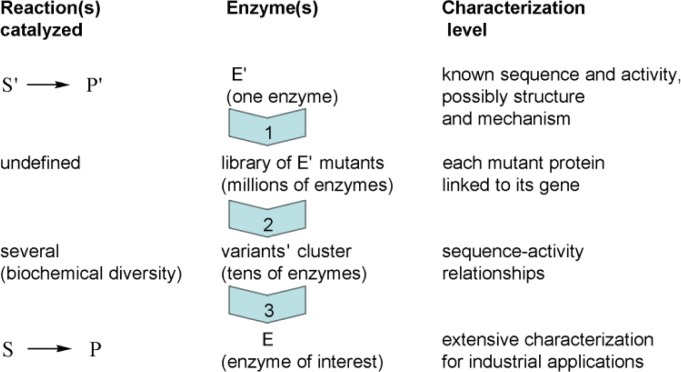
To isolate an enzyme E of interest catalyzing the synthesis of a product P, the method starts with the gene encoding an enzyme E’ catalyzing the synthesis of a similar product P’ by conversion of a substrate S’. The prime indicates the similarity between products P and P’, substrates S and S’ which may differ by one or several chemical group(s) and enzymes E and E’ which differ by one or more amino acid(s). The directed enzyme evolution experiment aims at the identification of the amino acid substitutions in E’ which are sufficient for catalysis of the conversion of S into P by enzyme E (of unknown sequence initially). The enzyme E’ found in nature does not catalyze the conversion of S into P. Optimization of E’ into E which catalyzes the reaction of interest is carried out by evolutionary increments using selection of few variants out of a large repertoire of enzymes. In step 1, the library of mutants is obtained after mutagenic amplification of the gene coding for E’. In step 2, a selection according to catalytic activity yields few enzymes of interest. If necessary, several cycles of selection and amplification of the corresponding genes provide the selected population of interest containing the variants of interest. In step 3, the several variant enzymes are characterized by their sequence, their expression level and their catalytic efficiency for the reaction of interest, thereby allowing enzyme E to be identified.

Catalysts used for the conversion of chemical products are typically organometallic compounds or enzymes [1–4]. Enzymes which represent most biocatalysts are of special interest as they are active in aqueous solutions instead of organic solvents, thereby minimizing waste and pollutants on the scales of tons for products of commercial interest: enzymes provide thereby an advantage over organometallic compounds as their use can be conceived within a sustainable development scheme at industrial scales. Enzymes are used on large scales such as in detergents and in numerous processes to make for example paper, drugs or food [3]. Importantly, enzyme- catalyzed reactions generally satisfy the twelve principles of green chemistry [5]. Selection then appears as the ideal tool to adapt, modify or optimize by incremental evolutionary steps an enzyme for the specific process satisfying industrial requirements (Scheme 1).

In this review, a general approach for the identification of catalysts is described: it makes use of the display of proteins on the surface of filamentous phages and of the coupling of products on phage in the proximity of enzymes that catalyze the substrate to product conversion (Scheme 1). This review does not aim to provide a general overview of the enzyme engineering field. It makes use of highly diverse fields from thermodynamics to microbiology to focus on a general method for the isolation of genes encoding catalysts.

## On selections of proteins according to catalytic activity

**Figure 1:**
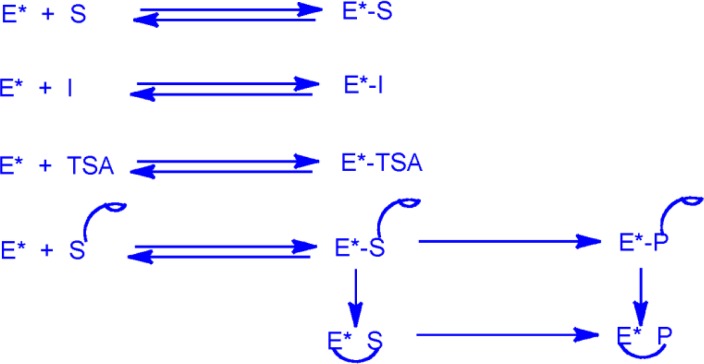
**Selections according to catalytic activity based on affinity selections**. Selections according to catalytic activity were carried out by affinity selections for enzyme-substrate complexes, for enzyme-inhibitor complexes, for complexes between enzymes and transition-state analogues or for complexes between enzymes and their reaction products. The asterisk indicates a diverse population of proteins such as millions of variant enzyme molecules. A hyphen indicates a complex between two molecules. The curved line indicates a covalent bond the enzyme and the substrate or the product. The curved line bound to a half-moon indicates a cross-linking group bound to the substrate or to the product. E = enzyme. S = substrate. I = inhibitor. TSA = transition-state analogue. P = product.

Because selections of proteins for binding to a target have now been done for several decades [6–8], it was found to be useful to design selections for catalysis as selections for binding (Figure 1) [9–12]. In the case of catalytic elution, the selection of enzymes is rather based on unbinding [13].

Selections of proteins for binding to a substrate make sense in the case of reactions catalyzed by an enzyme which forms a covalent intermediate with the substrate. Formation of a covalent bond with a substrate allows the isolation of proteins binding the substrate. This strategy does not provide a general means to isolate catalysts for the conversion of substrates into products, even though the covalent protein-substrate intermediate may mimic a transition-state for the reaction.

Suicide-inhibitors were also found of interest because stable covalent bonds can be formed between proteins considered as potential catalysts and a substrate mimic used for the isolation of these proteins. Suicide inhibitors cannot be designed for most chemical reactions and their synthesis is often time-consuming.

Transition-state analogues raised much interest because a higher stabilization of a transition-state than the stabilization of a substrate provides a way to decrease the activation energy, thereby defining a catalyst. Transition-states cannot be isolated because they correspond to energy maxima. Transition-state analogues can however be designed and generally synthesized. The fact that few catalysts were obtained using this approach may be caused by the approximation of transition-states by transition-state analogues. Further, proteins binding a transition-state analogue may not necessarily be adapted to bind a substrate and to release the reaction product as required within a catalytic cycle.

Intra-molecular or intra-complex catalysis provides a strategy to link reaction products to the proteins that catalyzed substrate to product conversion. Affinity chromatography for the product is then sufficient to isolate the enzyme that catalyzed the chemical reaction of interest [14]. Given that intra-molecular catalysis is favored over inter-molecular catalysis, this strategy can be adapted to populations of distinct protein molecules within a single aqueous solution in a unique reaction vessel. So as to characterize a single enzyme molecule isolated by affinity chromatography for the product bound to the enzyme, it is essential to link each potential catalyst to its corresponding gene : the unique gene can indeed be amplified and further sequenced for unambiguous characterization of the catalyst of interest.

## Linking each enzyme molecule to its gene: on directed protein evolution

While chemistry generally involves the experimental manipulation of a large number of identical molecules linked to the Avogadro number, the number of molecules per mole, systems have been designed in biological chemistry so as to handle experimentally large populations of diverse molecules such as millions or billions of distinct proteins, by linking them to their corresponding genes. Isolation of rare protein molecules of interest by selection among a large population of diverse proteins (the protein library) allows the characterization of rare catalysts by amplification and sequencing of the corresponding genes.

Several complementary methods allow a link between gene and enzyme to be created [15–18].

In nature, proteins and the corresponding genes are linked because of cell membranes which define the limit between the environment and the evolving unit, the cell. Protein evolution, such as antibody evolution can occur on short time scales such as a few weeks as well-known for the adaptative immune system of Vertebrates in response to infection by micro-organisms.

In cell display, a protein is similarly fused to a membrane protein for expression on the surface of the cell, the corresponding gene being located within the cell on a chromosome or on a plasmid.

In phage display, the protein is fused to a bacteriophage's capsid protein for expression on the surface of the viral particle; the corresponding gene is located within the capsid on the genetic material carried by the viral particle [19]. In the case of filamentous bacteriophages (*Inovirus*), which are non-lytic phages, the growth of their hosts (*Escherichia coli* bacteria) is not prevented by the bacteriophages which are extruded from slowly growing bacterial cells. The viral genetic material, a single-stranded DNA, and the viral proteins are synthesized by the metabolic machinery of the host prior to assembly in the bacterial periplasm and extrusion of the phage particles from the host. *In vivo* expression of the fusion proteins allows large proteins of more than 100 kDa to be displayed on phage [20].

In ribosome display, *in vitro* transcription and translation systems have been used to link messenger RNAs (mRNAs) to the corresponding proteins by stabilizing a mRNA-protein-ribosome complex. By reverse transcription, mRNAs are copied into copy DNAs (cDNAs) which can be further amplified by the polymerase chain reaction (PCR) for DNA sequencing, so as to obtain the sequence corresponding to the catalyst of interest.

In mRNA display, covalent bonds have been engineered between a mRNA and the corresponding protein, providing thereby a minimal size for the system comprising a genetic material and its corresponding protein.

A link is then created between each protein molecule and its encoding nucleic acids: the libraries of 10^5^ to 10^13^ proteins linked to their coding sequence are manipulated within a single tube.

**Figure 2:**
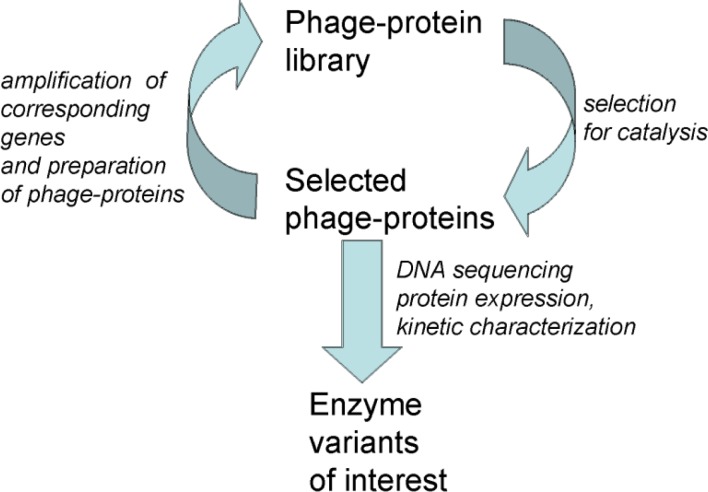
**Directed enzyme evolution.** A library of 10^6^ to 10^11^ distinct phage-protein variants is submitted to a selection for catalysis of the chemical reaction of interest. The phage-enzymes isolated contain the corresponding genes which are amplified by infection of *E. coli*. A secondary library of phage-proteins enriched into phage-enzymes is constructed and submitted to a further selection for catalysis. Cycles of selection for catalysis, amplification of the corresponding genes and preparation of the phage-protein library are carried out. The number N of cycles depends on the enrichment factor f per selection and on the size s of the library. It can be estimated for specific systems in directed enzyme evolution experiments (cf. text). Once the selected polyclonal population of phage-proteins is sufficiently enriched into active enzymes, the characterization of individual variants including sequencing of the corresponding genes, expression of the variant proteins and kinetic characterization of the catalysts is undertaken.

In directed enzyme evolution experiments, the protein library is then submitted to a selection according to catalytic activity so as to enrich the library into catalysts for the reaction of interest (Figure 2).

Experimentally, the enrichment factor f is easily measured for selections using model libraries consisting of two proteins such as an active enzyme and an inactive mutant: the enrichment factor f is then defined as the ratio between the proportion of active enzymes after selection and the proportion of active enzymes before selection. The higher the enrichment factor, the more efficient is the selection. For large libraries, a single selection is typically not sufficient to isolate the best catalysts. Cycles of protein selection for catalysis, amplification of the corresponding selected genes and preparation of secondary protein libraries are therefore iterated. The final enrichment factor for a directed enzyme evolution experiment is the product of the enrichment factors at each cycle. For a protein library of size s and for an enrichment factor f per cycle, the number N of cycles required for the directed evolution experiment can be estimated as:

N≅log(s)log(f)

For a number of cycles less than N, it cannot be expected to isolate the best catalysts by random sampling of several clones from the selected population of variants. For a number of cycles higher than N, a successful directed enzyme evolution experiment will typically yield by random sampling of several clones, the best catalyst(s) for the chemical reaction of interest.

Iteration of the selection and amplification cycles minimizes the residual background in selections but may also increase biases such as those due to the amplification step. To prevent significant biases within directed evolution experiments, it can be useful to introduce new or different types of selections for the isolation of variants of interest [21]. To reduce the need for time-consuming experiments during directed enzyme evolution cycles, the design of *in vivo* selections according to catalytic activity [14] and of continuous evolution procedures [22] is of major interest. It remains however a challenge to design such selections and procedures that could be adapted to most chemical reactions.

## Linking the catalytic efficiency of an enzyme molecule to its gene: on the chelate effect and its interpretation in thermodynamics

**Figure 3:**
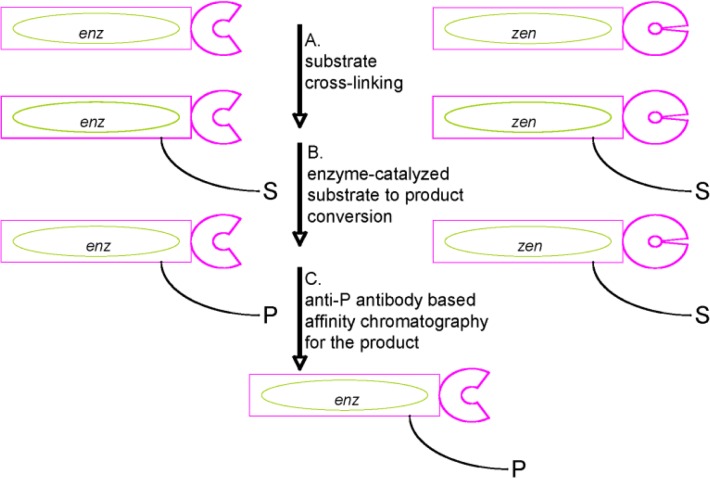
**Principle of a selection of proteins according to catalytic activity.** The principle is highlighted here for two proteins, an active enzyme coded the gene *enz* and an inactive protein variant coded by the gene *zen*. Both proteins are displayed on phage. This principle can be straightforwardly extended to libraries of millions of protein variants. In step A, library phage particles are cross-linked with the substrate. In step B, intramolecular catalysis occurs: the substrate bound to enzymes at proximity of the active site is converted into products bound to the phage particles. Substrates bound to inactive protein mutants are not converted into products and remain bound to the phage particles. In step C, affinity chromatography for the product using an anti-product antibody (anti-P) allows the specific isolation of active phage enzymes and of the corresponding genes. Steps A to C result in the selection of active enzymes and their genes from a library of active and inactive proteins displayed on phage [24]. The protein sequence of unique catalysts is then obtained by amplification and sequencing of the corresponding genes located within the bacteriophage particles.

The *in vitro* selection of proteins according to their catalytic activity is based on the intramolecular conversion of a substrate to the product crosslinked to the phage particle (Figure 3). Selections for catalysis ensure ideally that the complete catalytic cycle is carried out by the enzyme: binding of a substrate to the enzyme's active site, conversion of substrate into product and release of the product from the enzyme's active site.

**Figure 4:**
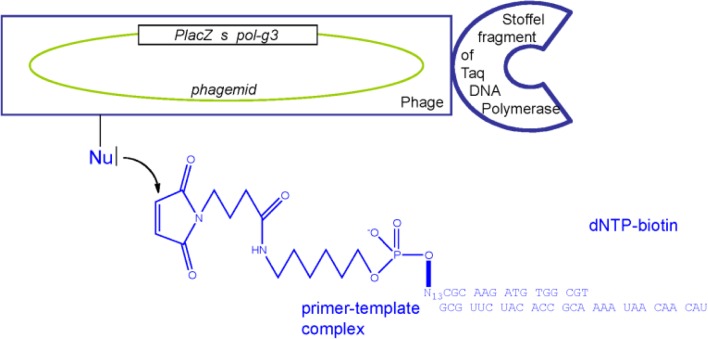
**A selection of proteins according to reverse transcriptase activity.** Variant enzymes such as DNA-polymerases are displayed on the surface of filamentous bacteriophages (*Inovirus*) as fusions with minor coat protein p3 located at one tip of the particle. The corresponding gene fusion is a single-stranded DNA fragment on the phagemid within the phage particle. *pol-g3* is the gene fusion coding for the enzyme variant fused to protein p3. *s* is the signal sequence for export of the fusion protein in the periplasm of *E. coli* prior to assembly of the phage particles in the presence of a helper phage [21]. *PlacZ* is the lacZ promoter allowing transcriptional activation in the presence isopropyl-beta-thiogalactoside in the bacterial culture medium and transcriptional repression in the presence of glucose in the culture medium. One substrate of DNA-polymerases is a deoxynucleotide triphosphate, which is derivatized by biotin on position 5 of the pyrimidine for further use in affinity chromatography to prevent any interference with the polymerization reaction. The other substrate of DNA-polymerases is a duplex of nucleic acids consisting of a primer hybridized to a template. So as to select for reverse transcriptase activity, the template is an RNA strand while the primer is an oligodeoxynucleotide. For the purpose of selection, the primer was derivatized at its 5’ end by a maleimide group known to form covalent bonds with nucleophiles (Nu) such as sulfhydryl groups if a cysteine in its reduced state is present on the phage surface or with primary amines such as protein amino termini or lysine side-chains. N_13_ are thirteen nucleotides [24].

This *in vitro* selection principle has been applied in the case of DNA-polymerases for the isolation of thermostable RNA-dependent DNA-polymerases (reverse transcriptases) (Figure 4).

If the variant enzyme is an active reverse transcriptase, biotinylated deoxynucleotide triphosphates are added at the 3’ end of the primer whose 5’ end is bound to the phage particle (Figure 4) [23]. If the variant protein is an inactive enzyme, no deoxynucleotide triphosphate is added at the 3’ terminus of primers; the primer-template complex cross-links the phage particle which is not labelled by biotin. Affinity chromatography for biotin using streptavidin-coated beads then allows the isolation of biotinylated phages displaying an active reverse transcriptase and containing within the particles the corresponding genes which can be amplified and further sequenced for characterization of active enzyme variants [9, 24].

**Figure 5:**
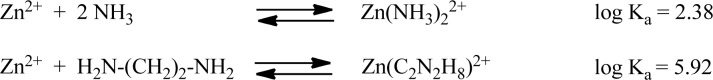
Association constants for complex formation between a zinc dication and a diamine ligand or ammine ligands at 25°C.

The chelate effect was first defined as the logarithm of the ratio between the association constant for complex formation between a metal cation and one diamine ligand and the association constant for complex formation between the same metal cation and two ammine ligands (Figure 5). It was theoretically interpreted as an effect which can be linked to the entropic term, but not to the enthalpic term of the free energy variation (ΔG = ΔH – T.ΔS) for a chemical reaction [26].

**Figure 6:**
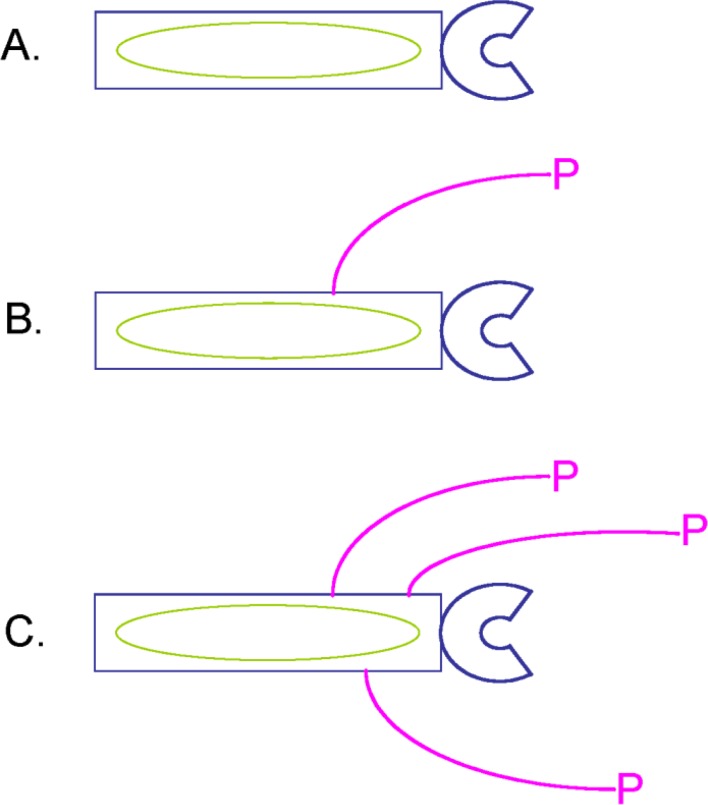
The higher the catalytic turnover, the larger the number of products bound to phage, the higher the avidity for a product-binding matrix. An extension of the chelate effect to large macromolecular assemblies such as a bacteriophage particle is used to select the protein variants with the highest catalytic activities. A. The protein displayed on phage has no catalytic activity of interest and the phage particle is not labelled by the product. B. The protein displayed on phage is catalytically active for the reaction of interest. Substrate to product conversion by the enzyme as well as cross-linking to phage yields a bacteriophage particle which is labelled by a product P. C. The enzyme variant displayed on phage has a higher catalytic activity than the protein variant B. More products are therefore bound to phage C than to phage B: intra-molecular cross-linking and catalysis within the phage particle are favoured over inter-molecular cross-linking and catalysis involving several phage-proteins of the library because of the chelate effect, provided there is no steric hindrance induced by the linker. Phage capture by binding to the product of interest is more efficient for phage C than for phage B, again because of the chelate effect. Phage A is not recovered specifically by binding to the product of interest and represents in the directed enzyme evolution experiment a background which can be eliminated by iterated cycles of selection for catalysis, amplification of the selected genes and production of the corresponding phage-proteins. Given that the DNA sequence of the protein variant is located within the phage particle, selection of the phage-protein variant with the catalytic activity of interest allows retrieval of the corresponding gene by amplification and sequencing [24].

The chelate effect provides the basis to distinguish the two concepts, avidity and affinity. While a product binds a target with a given affinity, a particle bound to multiple products binds the target with a higher avidity than the particle bound to a single product. Accordingly, a phage-enzyme with a high catalytic efficiency bound to multiple products released from the enzyme's active site will be isolated more efficiently by affinity chromatography for the product than a phage-enzyme with a low catalytic efficiency, which is not bound to any reaction product or bound to a single product which is not necessarily released from the active site (Figure 6).

## On relationships between sequences, structures and functions: the biochemical diversity in selected populations of variants

The biochemical diversity in the population of variants selected according to their catalytic activity is highlighted by the relationships between enzyme sequences and functions which are typically characterized by their kinetic parameters [24, 27]. Sets of functionally related enzyme variants are characterized at the sequence level by their genetic variability and define molecular quasi-species [28].

Enzyme variants can differ by few mutations and can be ordered in different enzyme classes as defined by the Enzyme Commission (EC) depending on their function(s). This suggested a bidimensional classification of enzyme classes highlighting functional clusters corresponding to different enzyme classes associated to highly similar sequences of closely related variants [29].

Genome annotations, which are the predicted biochemical functions associated to protein coding genes, are commonly based on sequence comparisons by homology and sequence alignments [30]. The molecular quasi-species and functional clusters identified for enzymes shall be extremely useful for the improvement of genome annotations as well as to provide hints for *in vivo* validation of biochemical functions [31].

Characterization of the variants’ sequence-function relationships are of special importance in the case of enzymes of industrial interest. As an example, reverse transcription coupled to the polymerase chain reaction appears as a powerful tool for the detection of RNA viruses in molecular diagnostics. For the design of thermostable reverse transcriptases (RNA-dependent DNA-polymerases), the directed enzyme evolution experiment started from the gene encoding *Thermus aquaticus* DNA polymerase I (Taq) known for its DNA-dependent DNA-polymerase activity and whose scaffold is thermostable. The library of mutant Taq DNA-polymerases constructed was submitted to cycles of selection according to RNA-dependent DNA-polymerase activity. Given the known structure of the complex between the DNA-polymerase and its substrates and the large interface between the template and the enzyme, the number and the nature of mutations required to improve reverse transcriptase activity could not be predicted. Increase by more than two orders of magnitude of the reverse transcription rate constants k_cat_ were measured for the directed evolution experiment in single turnover kinetic analyses according to the Michaelis-Menten model [24]. The selected population of thermostable reverse transcriptases provided sequence-function relationships, thereby allowing the biochemical diversity to be described and industrial applications to be devised in medical diagnostics [23].

## Conclusions

The strategy for the isolation of genes encoding catalysts described has several advantages:

- it was successfully applied for the engineering of thermostable RNA-dependent DNA-polymerases of interest in molecular diagnostics by reverse transcription and polymerase chain reaction.
- the high-fidelity synthesis of large proteins of more than 100 kDa displayed on phage by *in vivo* translation in *E. coli*.
- the wide spectrum of *in vitro* conditions for the selection according to catalytic activity, which does not necessitate an intracellular medium as used for *in vivo* selections and which can be ideally adapted for the chemical reaction of interest, because of the high resistance of the bacteriophage particles to chemical and physical denaturation.
- the selection for the highest catalytic efficiencies by making use of the chelate effect.
- the adaptability of the strategy to most types of chemical reactions such as synthetic reactions [24], substrate-cleaving reactions [25] as well as isomerizations by affinity chromatography for the reaction product allowing the parallel processing of 10^6^ to 10^11^ distinct protein variants.

The directed enzyme evolution strategy described herein allows the identification of sequence-activity relationships for catalysts. It relies on a unique combination of specific fields in the different natural sciences ranging from physical chemistry to microbiology through organic chemistry, molecular biology and biological chemistry. It highlights at the molecular level the power of Darwin's principles on evolution to answer a long-standing problem in chemistry: how to find catalysts for a given chemical reaction? By considering the gene of an enzyme catalyzing a similar chemical reaction and by directing its evolution.

**Table:** Advantages and disadvantages, requirements and limitations of screening and selection methods for the identification of biocatalysts

Criteria	Requirements, limitations, advantages and disadvantages
Processing of catalysts	-in parallel: single molecules assayed -simultaneously in selections -in serie: fraction of moles of molecules assayed in screenings
Quantity of substrate	-milligram scale for a selection -10 grams scale for a screening
Synthetic steps for substrate synthesis	-numerous for transition-state analogue synthesis -few for substrate modified by biotin, fluorescent groups or cross-linkers
Types of selections according to catalysis	selections by affinity - for transition-state analogue -for products bound to the enzymes catalyzing the reaction
Conditions for the selections according to catalytic activity	-wide range of buffers and temperatures for *in vitro* selections -intracellular medium as buffer for *in vivo* selections: incompatibility of compartmentation with buffer changes and addition of substrates
Selection for catalytic efficiency	multiple catalytic cycles assayed by linking products to genes encoding catalysts - within a cell with growth advantages (*in vivo* selection) -by affinity chromatography and using the chelate effect (*in vitro* selection)
Biocatalyst size	< 70 kDa in ribosome display and mRNA display < 140 kDa in phage display
Biocatalyst repertoire size	< 10^8^ in screening < 10^12^ in phage display < 10^14^ in ribosome display and mRNA display
Protein synthesis quality	-characteristic of *in vitro* translation systems in ribosome display -characteristic of *E. coli* translation in phage display
Post-translational modifications	-adjustable for *in vitro* translation systems *-E. coli* modifications adjustable in vitro in phage display -eukaryotic modifications in yeast display
